# Corrigendum: Interaction between habits as action sequences and goal-directed behavior under time pressure

**DOI:** 10.3389/fnins.2024.1393595

**Published:** 2024-04-09

**Authors:** Sascha Frölich, Marlon Esmeyer, Tanja Endrass, Michael N. Smolka, Stefan J. Kiebel

**Affiliations:** ^1^Department of Psychology, Technische Universität Dresden, Dresden, Germany; ^2^Department of Psychiatry, Technische Universität Dresden, Dresden, Germany; ^3^Centre for Tactile Internet with Human-in-the-Loop (CeTI), Technische Universität Dresden, Dresden, Germany

**Keywords:** habits, dual-process theory, response conflict, action sequence, goal-directed behavior, automatic behavior

In the published article, there was an error. The reported results for the repeated measures ANOVA on the high reward-probability choice frequencies in section 3.3 were incorrect. The results stated a significant main effect of day, which was incorrect. A correction has been made to section 3.3. Increase of sequence impact on day two, 2nd paragraph. The first sentence previously stated:

“A repeated measures ANOVA on the high reward-probability choice frequencies yielded a significant main effect of choice trial type [F_(1, 99)_ = 77.8, *p* < 0.0001, partial η^2^ = 0.12], a significant main effect of day [F_(1, 99)_ = 194.6, *p* < 0.0001, partial η^2^ = 0.36], and a significant interaction between these two factors [F_(1, 99)_ = 77.8, *p* < 0.0001, partial η^2^ = 0.12].”

The corrected paragraph appears below:

“A repeated measures ANOVA on the high reward-probability choice frequencies yielded a significant main effect of choice trial type [F_(1, 99)_ = 201.7, *p* < 0.0001, partial η^2^ = 0.46], no main effect of day, and a significant interaction between these two factors [F_(1, 99)_ = 94.7, *p* < 0.0001, partial η^2^ = 0.1]. This main effect of choice trial type can be readily seen in Figure 3, where participants generally chose the response option with the high reward probability more often in congruent than in incongruent trials. This reflects the relative increase of this choice difference from day one to day two. In random blocks, in the absence of a repeating action sequence, the frequency of high reward-probability choices increases from day one to day two and can be interpreted as a baseline without sequence influence. On both days, participants chose the high reward-probability response more often in congruent trials than in random choice trials (85.5% in congruent trials, 79.6% in random choice trials on day one, *p* < 0.0001, Cohen's *d* = 0.62 day one, 90.7% in congruent trials and 84.0% in choice trials on day two, *p* < 0.0001, Cohen's *d* = 0.86, two-tailed paired *t*-tests) and less often in incongruent trials than in random choice trials (75.0% in incongruent trials on day one, *p* < 0.0001, Cohen's *d* = −0.39, and 68.1% on day two, *p* < 0.0001, Cohen's *d* = 1.19, two-tailed paired *t*-tests). Participants seem to learn the action sequence quickly, with a difference in the high reward-probability choices between congruent and incongruent trials of 25.9% in the second half of the first sequential block, compared to 10.6% to the first half, and no difference within the first 40 trials of the first block.”

In the published article, there was also an error in Figure 5 as published.

The correlation plots between ΔRT and H.P. Choice Difference for days 1 and 2 were incorrect. The Pearson correlation on day 2 is stronger than previously reported (0.58 instead of 0.54). While we previously reported that the regression slope does not change from day 1 to day 2, it does in fact increase significantly (*p* = 0.03 instead of *p* = 0.1).

The corrected Figure 5 and its caption appear below.

**Figure 5:**
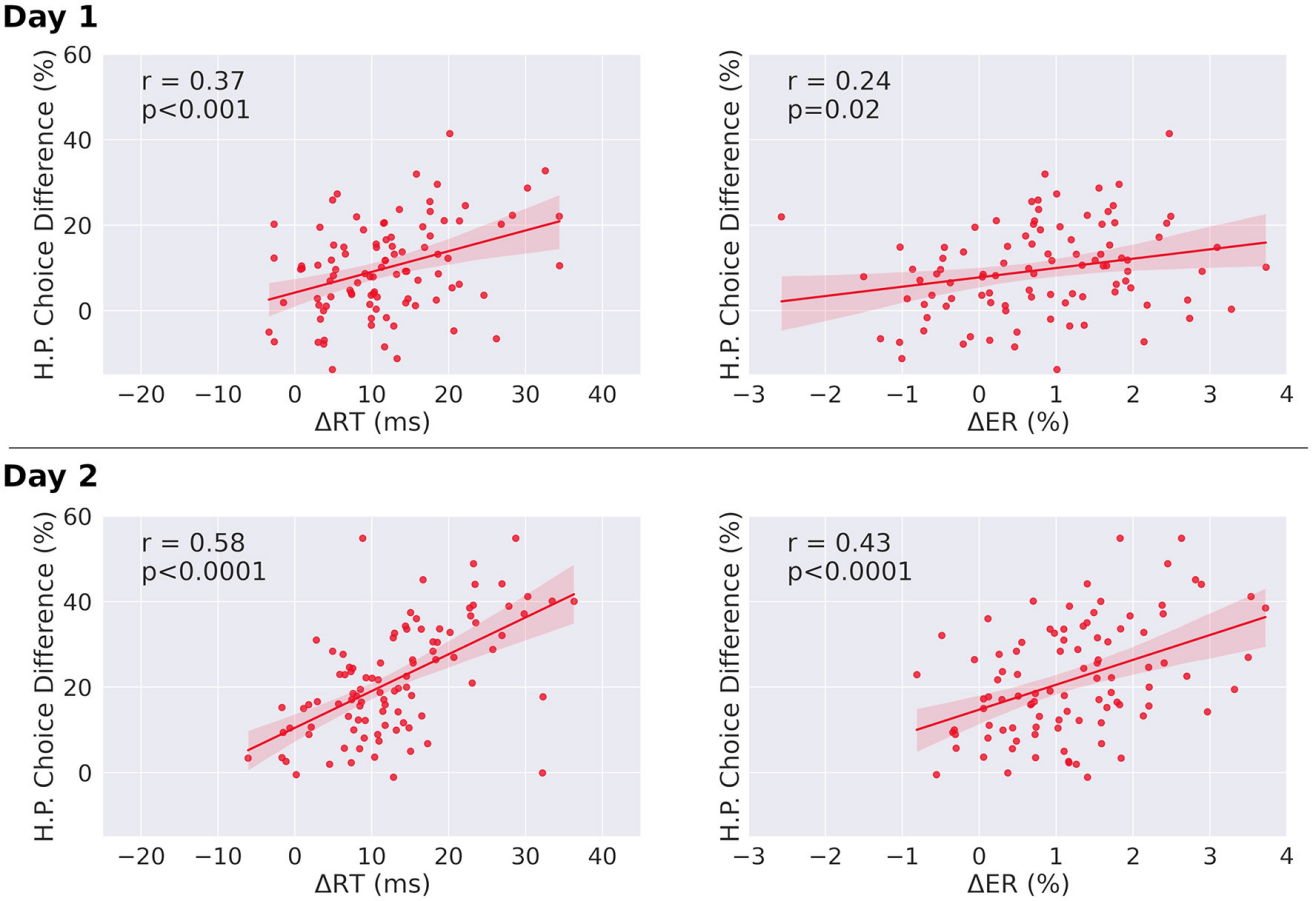
Correlations between choice differences and both reaction time differences and error rate differences, in single-target trials. **(Left)** Correlation of individual H.P. choice frequency for congruent and incongruent trials (H.P. choice difference) and mean reaction time differences between sequential and random condition for single-target trials. Both slopes are significantly different from zero. The correlation coefficient of day two is significantly increased relative to day one (*p* < 0.0001). **(Right)** Correlation of individual H.P. choice frequency for congruent and incongruent trials (H.P. choice difference) and mean error rate differences between sequential and random condition for single-target trials. Both regression slopes are significantly different from zero. The correlation coefficient of day two is significantly increased relative to day one for both error rates and reaction times (*p* < 0.0001). The regression slope further increases from day one to day two for error rates (day 1: slope = 2.2, day 2: slope = 5.8, p_difference_ = 0.018), and for reaction times (day 1: slope = 0.48, day 2: slope = 0.86, p_difference_ = 0.03). Δ*RT* = *RT*_Random_ – *RT*_Seq_, Δ*ER* = *ER*_Random_ – *ER*_Seq_. Analysis was performed after outlier removal (outliers were defined as elements more than three scaled median absolute deviations from the median). Results before outlier removal are similar for reaction times. For error rates, before outlier removal, the correlation is not significant for day one (*r* = 0.16, *p* = 0.12), but significant for day two (*r* = 0.40, *p* < 0.0001).

The authors apologize for these errors and state that this does not change the scientific conclusions of the article in any way. The original article has been updated.

